# Astaxanthin from *Haematococcus pluvialis* Prevents Oxidative Stress on Human Endothelial Cells without Toxicity

**DOI:** 10.3390/md13052857

**Published:** 2015-05-07

**Authors:** Philippe Régnier, Jorge Bastias, Violeta Rodriguez-Ruiz, Noelia Caballero-Casero, Carmen Caballo, Dolores Sicilia, Axelle Fuentes, Murielle Maire, Michel Crepin, Didier Letourneur, Virginie Gueguen, Soledad Rubio, Graciela Pavon-Djavid

**Affiliations:** 1 INSERM U1148, Laboratory for Vascular Translational Science; University Paris 13, PRES Sorbonne Paris Cité 99, Av. Jean-Baptiste Clément, 93430 Villetaneuse, France; E-Mails: philregnier@hotmail.fr (P.R.); violeta.rodriguezruiz@univ-paris13.fr (V.R.-R.); axelle.fuentes@gmail.com (A.F.); murielle.maire@univ-paris13.fr (M.M.); michel.crepin@inserm.fr (M.C.); didier.letourneur@inserm.fr (D.L.); virginie.gueguen@univ-paris13.fr (V.G.); 2 CORDUNAP IBT, Avenida Playa Brava 3256, 1100000 Iquique, Chile; E-Mail: jorge.bastias@cordunap.cl; 3 Department of Analytical Chemistry, Institute of Fine Chemistry and Nanochemistry, Campus of Rabanales, University of Córdoba, 14071 Córdoba, Spain; E-Mails: noelia.caballero.c@gmail.com (N.C.-C.); calic_amb@hotmail.es (C.C.); qa1sicrm@uco.es (D.S.); qa1rubrs@uco.es (S.R.)

**Keywords:** astaxanthin, antioxidants, *Haematococcus pluvialis*, oxidative stress, HUVEC, TEAC

## Abstract

Astaxanthin, a powerful antioxidant, is a good candidate for the prevention of intracellular oxidative stress. The aim of the study was to compare the antioxidant activity of astaxanthin present in two natural extracts from *Haematococcus pluvialis*, a microalgae strain, with that of synthetic astaxanthin. Natural extracts were obtained either by solvent or supercritical extraction methods. UV, HPLC-DAD and (HPLC-(atmospheric pressure chemical ionization (APCI)+)/ion trap-MS) characterizations of both natural extracts showed similar compositions of carotenoids, but different percentages in free astaxanthin and its ester derivatives. The Trolox equivalent antioxidant capacity (TEAC) assay showed that natural extracts containing esters displayed stronger antioxidant activities than free astaxanthin. Their antioxidant capacities to inhibit intracellular oxidative stress were then evaluated on HUVEC cells. The intracellular antioxidant activity in natural extracts was approximately 90-times higher than synthetic astaxanthin (5 µM). No modification, neither in the morphology nor in the viability, of vascular human cells was observed by *in vitro* biocompatibility study up to 10 µM astaxanthin concentrations. Therefore, these results revealed the therapeutic potential of the natural extracts in vascular human cell protection against oxidative stress without toxicity, which could be exploited in prevention and/or treatment of cardiovascular diseases.

## 1. Introduction

Oxidative damage is initiated by reactive oxygen species (ROS) and influences the pathogenesis of various disorders, such as atherosclerosis, neurodegenerative diseases, inflammatory diseases and aging. The mechanism of oxidative stress is an imbalance between ROS production and the antioxidant defense capacities of the cell, leading to reactions between those oxidative molecules and lipids, proteins and DNA [1,2].

Endothelial dysfunction is recognized as an early marker of atherogenic risk [3,4]. Physiologically, the respiratory chain in mitochondria produces small amounts of ROS. Under these conditions, free radicals are involved in the regulation of several processes, such as signaling pathways and gene expression, but they are also involved in the proliferation, migration and apoptosis of vascular cells [5]. In an overproduction of ROS (hypertension, hypercholesterolemia, ischemia/reperfusion, hyper oxygenation, stress, pollution, radiation, *etc*.), the physiological mechanisms of control and protection are overwhelmed, and ROS interact with nitric oxide (NO) to form peroxynitrite (ONOO^−^), a highly toxic compound. The bioavailability of endothelial NO is then reduced, and its vasoprotective effect is disrupted [6,7,8].

Antioxidant molecules, especially carotenoids, play an important role in the control of the oxidative process. These antioxidant molecules (carotenoids) possess a strong antioxidant power due to their double-bonded structure, allowing the delocalization of impaired electrons [9,10]. There is a growing interest in astaxanthin (3,3′-dihydroxy-β-β′-carotene-4,4′-dione), from the carotenoids’ family. It is a xanthophyll, meaning that, unlike β-carotene and lycopene, it is a polar molecule that is able to scavenge free oxygen radicals. The polyene chain in astaxanthin crosses the cell membrane, allowing the polar ends of the molecule to be exposed to the cytoplasm and external sides of the cell in the meantime [1,11]. This disposition facilitates the electron transfer from the cytoplasm to the outer part of the cell. The terminal ring of the astaxanthin seems to be the final scavenger of the ROS. The astaxanthin by its provision could also have a synergistic effect with vitamin C that would recharge astaxanthin once it has scavenged ROS [12]. One of the major applications of astaxanthin is its use as feed additive for salmonid fish species. The European Food Safety Authority recommends a dietary amount of synthetic astaxanthin of 100 mg/kg. This astaxanthin diet can be extended to other fish, ornamental fish and crustaceans at the same dose [13]. Moreover, its antioxidant properties confer to astaxanthin an interesting therapeutic potential with applications, such as anticancer, anti-diabetic and anti-inflammatory agent [1]. This molecule has shown promising results in animal, but also in human experiments, leading to a decrease of blood pressure and an increase of HDL rate [11,14,15,16]. When used in pre-conditioning, astaxanthin lessened the extent of myocardial infarction in rats and rabbits and thus appears very promising for cardiovascular treatment [17,18]. Oral administration of astaxanthin to ApoE and Low Density Lipoprotein-Receptor KO mice also reduced the occurrence of aortic atheroma [19] and, when tested on HUVEC and murine platelets, showed a decreased production of ONOO^−^ and increased NO bioavailability [20].

Natural astaxanthin may come from different origins, derived from algae, crustaceans or krill, and be extracted in many different ways [1,21]. Thus, synthetic astaxanthin (AstaS) is a racemic mixture of three isomers (3-*R*,3′-*R*), (3-*R*,3′-*S*) and (3-*S*,3′-*S*) [11,22], whereas the chemical composition of natural astaxanthin depends on the natural source and even on the extraction method applied. For instance, extracts of *Haematococcus pluvialis* (a reference microalgae strain for the production of natural astaxanthin) contains a mixture of carotenoids where an astaxanthin isomer (3-*S*,3′-*S*) and its ester derivatives are the major compounds [11]. Due to the lack of homogeneity in the composition of the natural source samples, a complete physicochemical study is mandatory. The purpose of this work was to carry out a comparative study between three astaxanthin samples: synthetic astaxanthin and natural astaxanthin from *Haematococcus pluvialis* extracted by two different methods. First, a physicochemical characterization of samples by spectrophotometry and HPLC-MS was performed. Next, their antioxidant activities were evaluated by using the Trolox equivalent antioxidant capacity (TEAC) and oxygen radical antioxidant capacity (ORAC) assays. Finally, a biological evaluation was carried out in order to assess their biocompatibility and their ability to inhibit intracellular stress in human endothelial cells for cardiovascular applications.

## 2. Results and Discussion

### 2.1. Physicochemical Characterization

#### 2.1.1. Astaxanthin Identification and Measurements by Spectrophotometry

**Figure 1:**
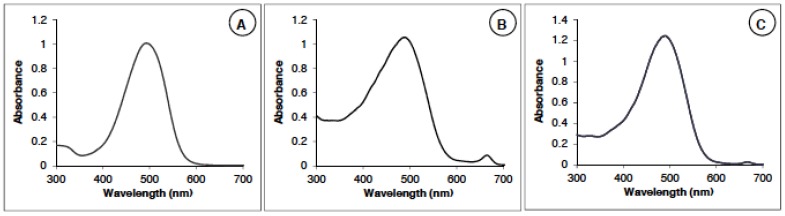
Spectra of AstaS (Asta, astaxanthin; S, synthetic) (**A**) and AstaP (P, powder) (**B**) and AstaCO_2_ (**C**).

Two natural extracts of dried powder of microalgae Haematococcus pluvialis were studied. The first one (called AstaP; Asta, astaxanthin; P for powder) was obtained by solvent (DMSO) extraction, and the second one (called AstaCO_2_) underwent a CO_2_ supercritical process. Final formulations of astaxanthin were presented as DMSO solution (AstaP) and sunflower oil solution (AstaCO_2_). Synthetic astaxanthin (AstaS) was used as the standard, showing a maximum of absorption at a wavelength of 482 nm (Figure 1A).

The absorption spectra of the AstaP (Figure 1B) and AstaCO_2_ (Figure 1C) have a main peak corresponding to astaxanthin at 482 nm and one small peak at 670 nm. This secondary peak corresponds to the chlorophyll present in the extract [23]. The absorbance in the region 300–400 nm is also larger than the one of the synthetic spectrum (AstaS) due to the absorption of other species (see Section 2.1.2).

#### 2.1.2. Characterization by HPLC-DAD and (HPLC-(APCI+)/IT-MS)

**Figure 2:**
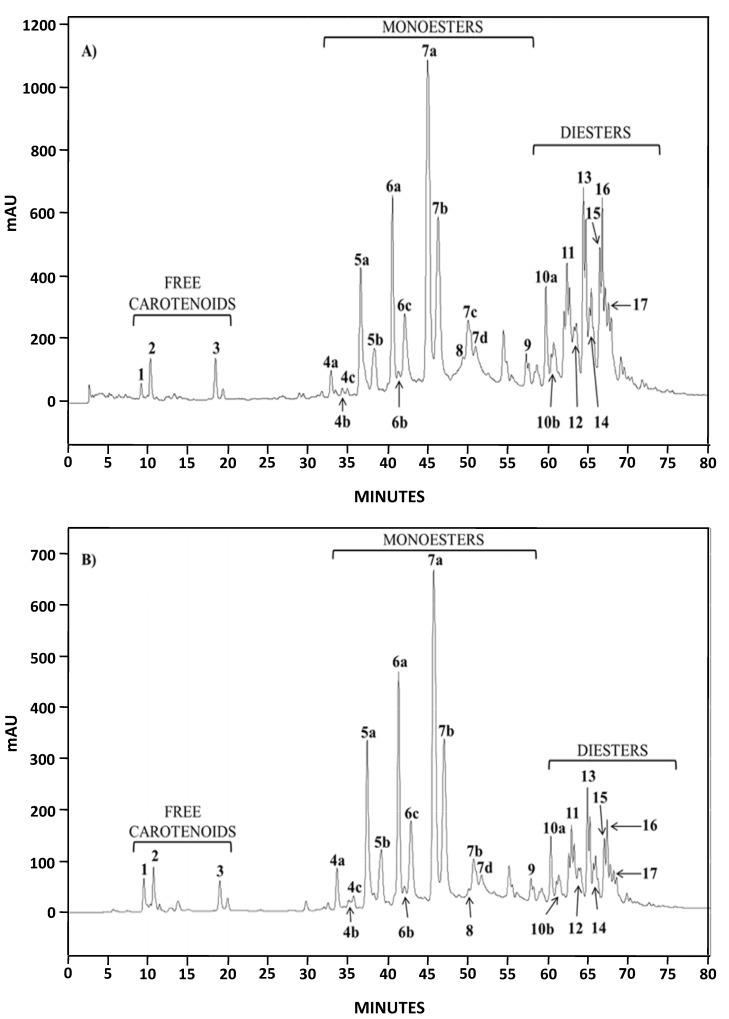
HPLC chromatograms (DAD, 482 nm) of AstaP (**A**) and AstaCO_2_ (**B**).

Both natural extracts (AstaP and AstaCO_2_) were characterized by liquid chromatography-diode array detection (HPLC-DAD) and HPLC-atmospheric pressure chemical ionization in the positive ion mode/ion trap mass spectrometry (HPLC-(APCI+)/IT-MS). The analysis of the chromatograms of AstaP and AstaCO_2_ by HPLC-DAD in the range 200–800 nm (data not shown) showed the presence of other compounds, all of them with two absorption peaks (330 and 400–450 nm). Moreover, in both spectrophotometry and HPLC analysis, the contents of these compounds were higher for AstaP. Compounds with absorption peaks around 400 nm in *H. pluvialis* extracts have been previously identified as astacin monoesters [24]. The chromatographic profile obtained for both extracts at 482 nm was almost identical (Figure 2) and similar to the one obtained by extracting *Haematococcus pluvialis* with petroleum ether [24].

**Figure 3:**
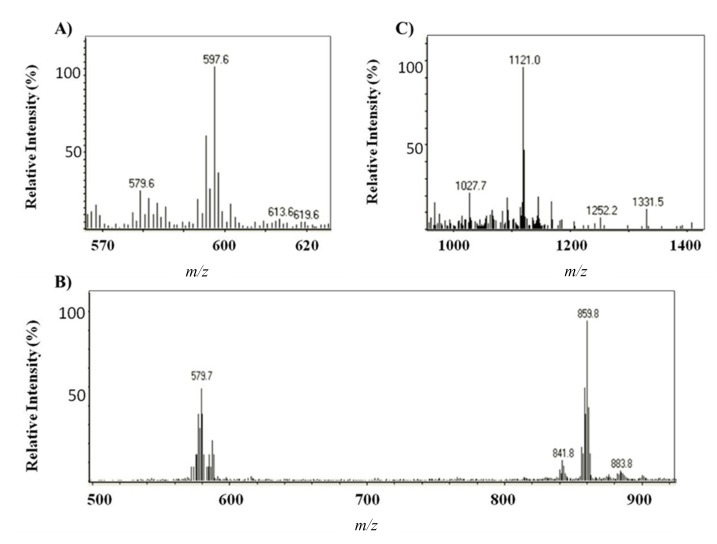
Mass spectra obtained by HPLC-APCI+ for (**A**) astaxanthin, (**B**) the astaxanthin monoester (ME) C_18:2_ and (**C**) the astaxanthin diester (DE) C_18:3_/C_18:2_ The assignment of respective ions is as follows: (**A**) *m*/*z* 597.6 ([M + H]^+^), *m*/*z* 579.6 ([MH − H_2_O]^+^); (**B**) *m*/*z* 859.8 ([M + H]^+^), *m*/*z* 579.7 ([MH − C_18:2_(280.1)]^+^; (**C**) *m*/*z* 1121.0 ([M + H]^+^).

Identification of major compounds in the microalga extracts was performed by HPLC-(APCI+)/IT-MS based on their molecular mass and characteristic fragmentation pattern [24]. The elution order was free carotenoids, astaxanthin monoesters and astaxanthin diesters, with retention times in the intervals 9–20, 32–59 and 60–75 min, respectively. Three free carotenoids were identified; astaxanthin, echinenone ([M + H]^+^ = 551.4) and canthaxanthin ([M + H]^+^ = 565.6) (Peaks 1–3, respectively, in Figure 3A,B) [24]; the former and the latter were also confirmed by the use of standards. The predominant fatty acids in astaxanthin monoesters were C_18:4_, C_18:3_, C_18:2_, C_18:1_, C_16:0_ and C_17:1_ (which corresponds to Peaks 4a–c, 5a–b, 6a–c, 7a–d, 8 and 9, respectively), whereas the main ones in astaxanthin diesters were C_18:3_, C_18:2_, C_18:1_, C_16:0_ and C_16:1_. Figure 3 shows, as an example, the mass spectra obtained for astaxanthin, the monoester (ME) C_18:2_ (Peaks 6a–c) and the diester (DE) C_18:3_/C_18:2_ (Peaks 10a–b).

The percentages of all forms of astaxanthin (free and esters) in AstaP and AstaCO_2_ were determined from relative area peaks, since similar absorption was obtained in the mobile phase of acetone. These percentages are shown in Table 1.

**Table 1:** Content of astaxanthin esters in the DMSO and CO_2_ extracts of *H. pluvialis*, expressed as a percentage of the total astaxanthin compounds found in each extract.

Chromatographic Peak Number	Compound	Content in AstaP (%)	Content in AstaCO_2_ (%)
1	Astaxanthin	0.45	1.70
4–9	Monoesters	62.6	76.1
10–17	Diesters	36.9	22.2

Although astaxanthin monoesters were predominant in both extracts, the distribution of free, monoesters and diesters of astaxanthin was quite different (Table 1, 0.45%, 62.6% and 36.9% for AstaP and 1.70%, 76.1% and 22.2% for AstaCO_2_). These results indicate that DMSO was more effective in extracting diesters and that approximately 3.8-fold more free astaxanthin was present in AstaCO_2_.

#### 2.1.3. Physicochemical TEAC Assay

The antioxidant activity of astaxanthin has often been compared to Trolox [25,26]. However, the source and composition of the samples are not often specified, and TEAC values can be expressed in different ways (slope ratios or mmol Trolox/g). This disparity makes the comparison between different extracts and the interpretation of the results very difficult.

Herein, the antioxidant activities of the three astaxanthin samples (synthetic astaxanthin (AstaS) and natural extracts (AstaP and AstaCO_2_) were studied and compared by the TEAC assay. This method correlates the antioxidant activity of carotenoids to their ability to neutralize 2,2′-azino-bis(3-ethylbenzothiazoline-6-sulphonic acid) diammonium salt radical cation (ABTS^•**+**^), determined by decolorization.

**Figure 4:**
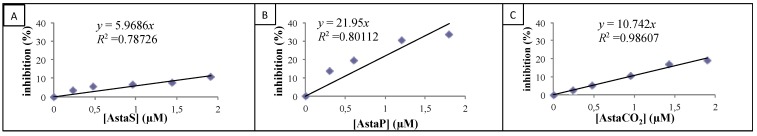
Inhibition of 2.2′-azino-bis(3-ethylbenzothiazoline-6-sulphonic acid) diammonium salt radical cation (ABTS^•**+**^) absorbance for various concentrations of AstaxS (**A**), AstaxP (**B**) and AstaxCO_2_ (**C**).

The dose-response curves of inhibition percentages for standard and astaxanthin extracts are presented in Figure 4A–C, and their slopes were determined.

Their TEAC values were then calculated by dividing these latter values by the experimental Trolox slope (4.5) (see Table 2).

**Table 2:** Antioxidant capacity of carotenoids evaluated by Trolox equivalent antioxidant capacity (TEAC) and oxygen radical antioxidant capacity (ORAC) methods. Each value is expressed as the mean ± SD (*n =* 3).

Product	TEAC			ORAC
	Unitless (Slope Ratio)	(mmol Trolox/g)	References	(µM TE)
AstaxS	1.32 ± 0.15	2.21 ± 0.25 ^a^	2.43 ^a^ [27]	1.68 ± 0.25
AstaP	4.37 ± 0.33	0.18 ± 0.01 ^b^	0.1–0.25 ^b^ [28]	8.1 ± 1.21
			0.1–0.4 ^b^ [27]	
AstaCO_2_	2.37 ± 0.11	3.01 ± 0.14 ^b^		4.07 ± 0.61

^a^ Millimoles Trolox/g AstaS; ^b^ mmol Trolox/g extract.

In order to compare these values with those found in the literature, the TEAC values were reported for the mass of the astaxanthin molecule or the extract. Results showed that for the same concentration, natural extracts (AstaP and AstaCO_2_) present stronger antioxidant activities than synthetic astaxanthin (AstaS) (see Table 2, Column 1). These results are consistent with those from Capelli *et al.* [29], which showed, by using a chemiluminescent method, that natural astaxanthin from *H. pluvialis* (extracted oleoresin) exhibited higher free radical scavenging power than the synthetic one.

Nowadays, the relative antioxidant capacity of the different astaxanthin forms present in natural sources (free astaxanthin, mono- and di-esters) is still debated. Some studies reported stronger antioxidant activity for free astaxanthin [27,30], whereas others indicated that esters (especially diesters) are more efficient than the free form [31,32]. As we already mentioned, synthetic astaxanthin is available as the free form of astaxanthin, but with their three stereoisomers, whereas the extracts contain only one isomer and esterified (mono- or di-) forms. Our TEAC results could be correlated to the composition determined by HPLC, and the hypothesis is that astaxanthin esters (predominant in AstaP and in AstaCO_2_; see Table 1) are more powerful as antioxidants than the free species.

#### 2.1.4. ORAC Assay

The antioxidant activity of astaxanthin samples against 2,2′-azobis(2-amidino-propane (AAPH) peroxyl radicals was chemically analyzed using the ORAC method. The ORAC test is a standard test based on the kinetic evaluation of hydrogen atom transfer-type reaction [33]. The decrease of the fluorescence intensity of fluorescein due to the action of AAPH was monitored during 1 h. The scavenging effect of samples was studied, and the antioxidant activity of AstaP, AstaS and AstaCO_2_ was evaluated as the capacity to retain the fluorescence of fluorescein in the presence of AAPH. A calibration curve of Trolox (reference antioxidant molecule) was drawn by plotting the area under the curve (AUC) *vs*. Trolox concentration (1–100 µM). The antioxidant activity of the samples was calculated relative to the Trolox activity, and the results are presented in Table 2, expressed as Trolox equivalent µM. The results showed that the antioxidant activity of synthetic astaxanthin is 1.6 µM TE. This value is in agreement with those published by Naguib [26] for commercial astaxanthin in concentrations of the same order of magnitude as those that we used. The antioxidant activity obtained by the ORAC test shows a different absolute value than that obtained by the ABTS test; however, we observe the same tendency of the results, namely AstaP > AstaCO_2_ > AstaS.

### 2.2. Biological Evaluation: Cellular Effects

#### 2.2.1. Cell Viability and Morphology

Cellular exposition to the antioxidants molecules may be subject to changes in cell morphology, the rate of cell growth or cell death. We monitored cell viability in the presence of natural extracts (AstaP and AstaCO_2_) and synthetic astaxanthin (AstaS) by the MTT test, previously described by Mosmann [34]. This method is based on the reduction of tetrazolium salt (MTT) by mitochondrial succinic dehydrogenases in viable cells, yielding purple formazan crystals [34].

**Figure 5:**
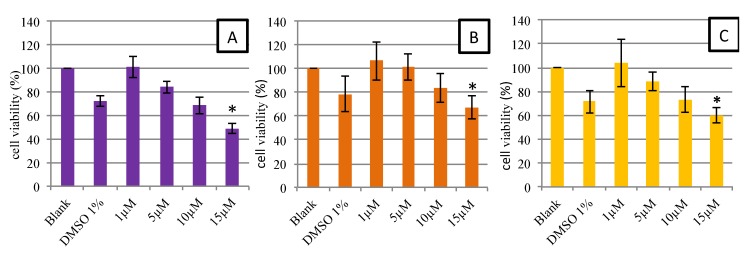
MTT assay: dose-response curves for cell viability assessed in human endothelial cells (HUVEC) exposed during 48 h to AstaS (**A**), AstaP (**B**) and AstaCO_2_ (**C**). Data are presented as the mean ± SD. * Significant toxicity with a cell population <70% of the blank (*n =* 3).

Figure 5 shows the viability of HUVECs after 48-h contact with different concentrations of synthetic and natural astaxanthin (1, 5, 10 and 15 µM). All final astaxanthin samples (AstaS, AstaP and AstaCO_2_) were prepared with up to 1% DMSO. Thus, 1% DMSO in the culture medium was tested. As shown in Figure 5, 1% DMSO in the culture medium led to a decrease in the viability of around 15%–20%. Nevertheless, the non-decrease of viability was observed in the 1 and 5 µM astaxanthin samples, suggesting a protective effect of astaxanthin. From 15 μM, a significant decrease in cell viability below the threshold of 70% was observed, regardless of the type of astaxanthin. This decrease in viability was not observed for the Trolox (data not shown). We can conclude that synthetic and natural astaxanthins are not toxic to HUVECs up to the concentration of 15 µM. These results were consistent with those of the literature, in particular with the study of Nagaraj *et al.* [35]. In this study, the authors performed the same protocol, but using HepG2 and MCF-7 cancer cells, which should be more resistant to toxic drugs. They showed that up to a dose of 10 μg/mL (16.75 μM), cell viability was maintained and that morphological changes and a significant decrease in viability were observed above a concentration of 15 μg/mL (25.13 μM).

**Figure 6:**
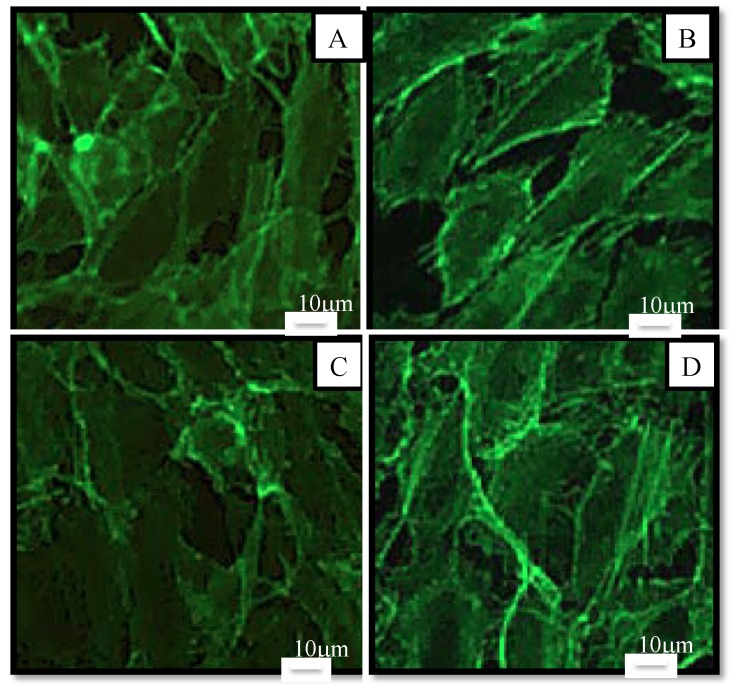
Morphology of human endothelial cells observed under fluorescence microscope after 48 h in culture medium (control (**A**)) or in samples at a concentration of 10 µM in culture medium (AstaS (**B**), AstaP (**C**) and AstaCO_2_ (**D**). Cells were stained with Alexa Fluor-conjugated phalloidin for detection of actin filaments. Scale bar: 10 μm.

To study the morphological effects at 10 µM (our maximum non-toxic concentration in MTT), cells were stained and observed by fluorescence microscopy (Figure 6). Results showed that there was no noticeable difference in morphology. Cells had the same elongated shape as the control. According to Nagaraj *et al.* [35], the mechanism of toxicity of astaxanthin is dual: an increase in the permeability of the membrane and an interaction with microtubules resulting in the blockage of the mitotic cycle for high concentrations (>40 µM). Therefore, the absence of morphological changes of HUVEC at 10 μM confirmed the absence of toxicity of astaxanthin at this concentration.

Moreover, we noticed that when AstaS or AstaP were added to the culture medium, a precipitate appeared after a few hours despite the previous filtering during the extraction process. By microscopy, we observed the formation of small solid deposits, which increased with the concentration of astaxanthin. In order to avoid this phenomenon, we then used a concentration of 5 μM astaxanthin for the following experiments.

#### 2.2.2. Cellular Antioxidant Activity on Endothelial Cells

There are very few examples in the literature where the intracellular antioxidant capacity of astaxanthin was evaluated. We set up our experiments based on the intracellular stress-based model (cellular antioxidant activity (CAA)) described by several groups [36,37,38]. We induced an *in vitro* oxidative stress in HUVEC cells using the *tert*-butyl hydroperoxide (Luperox, *t*-BuOOH). This molecule damages mitochondrial membranes leading to an intracellular overproduction of ROS [39,40]. In this method, Dichloro-dihydro-fluorescein diacetate DCFH-DA, an oxidation-sensitive indicator is used to measure ROS production in HUVEC cells. In the presence of ROS, DCFH is oxidized to fluorescent 2′,7′-dichlorofluorescein (DCF), which can be measured by fluorometry. If an antioxidant is present, it prevents the DCFH from being oxidized. The resulting fluorescence signal is lower, and thus, the CAA (which is inversely proportional) can be calculated (see the Experimental Section).

Samples were compared to each other at a concentration of 5 µM (Table 3) to avoid the formation of solid deposits in the culture medium (see Section 2.2.1). In accordance with the TEAC and ORAC tests, the results show that the antioxidant activities of natural extracts from microalgae AstaCO_2_ and AstaP were higher than AstaS.

**Table 3:** Comparison of cellular antioxidant activity at 1 h of samples at a concentration of 5 µM. Results are expressed in cellular antioxidant activity (CAA) % and presented as the mean ± SD. * Significant differences between the AstaS group and the AstaCO_2_ and AstaP groups.

Product	CAA (%)
AstaS	0.3 ± 0.2 *
AstaP	25.4 ± 9.5
AstaCO_2_	30.4 ± 12.7

Differences in CAA between the AstaP and AstaCO_2_ groups (25.4 and 30.4, respectively) were not statistically significant, whereas in TEAC tests, AstaP was found to have stronger antioxidant activity than AstaCO_2_ (4.8 and 2.3, respectively). This could be explained by factors affecting the CAA assay, such as the lipid/aqueous affinity of the molecule and the oxidation conditions [41]. These CAA results were also in agreement with the hypothesis already proposed of stronger antioxidant activities of ester forms present in natural extracts.

The results for natural extracts (AstaP and AstaCO_2_) are in accordance with those shown by Chang *et al.* [25]. The authors studied the antioxidant and neuroprotective effect of the natural astaxanthin (from Sigma) using the same fluorescent probe (DCFH-DA), but a different stress model (H_2_O_2_ instead of Luperox) and on a different cell line (rat pheochromocytoma (PC12) cells instead of HUVEC). In the same way, Guerra *et al.* [42] showed that a treatment with astaxanthin (2 µM, from Sigma, St. Louis, MO, USA) was able to partially reduce intracellular ROS production and restore the phagocytic capacity of human neutrophils. Thus, all of these results assert that natural astaxanthin plays a protective role in cells exposed to oxidative stress.

## 3. Experimental Section

### 3.1. Chemicals and Biological Reagents

Dimethyl sulfoxide (DMSO, Lot: SZBD1830V), 3-(4,5-dimethyl-2-thiazolyl)-2,5-diphenyl-2*H*-tetrazolium bromide (MTT), Isopropanol, 2′,7′-dichlorofluorescein 3′6′-diacetate (2′,7′-DCFH-DA, Lot: 092M4004V), *tert*-butyl hydroperoxide (*t*-BuOOH/Luperox Lot: BCBJ2885V), AAPH (2,2′-azobis(2-amidino-propane) dihydrochloride (Ref. 440914) and fluorescein (Ref. F6377) were purchased from Sigma-Aldrich (Saint-Louis, MO, USA). The same firm also provided 6-hydroxy-2,5,7,8-tetramethylchroman-2-carboxylic acid (Trolox, lot: BCBJ8170V) and 2,2′-azino-bis(3-ethylbenzothiazoline-6-sulphonic acid) diammonium salt (ABTS, lot: 061M538V). Acetone (HPLC gradient grade) was supplied by Panreac (Barcelona, Spain).

Concerning the cell culture, minimum essential medium (MEM), phosphate buffered saline (PBS), fetal calf serum and penicillin-streptomycin-amphotericin (PSA) were provided by GIBCO (Life Technologies, Carlsbad, CA, USA). Stable glutamine was purchased from PAA (PAA laboratories GmbH, Pasching, Austria). Human umbilical vein endothelial cells (HUVEC) were purchased from ATCC (CRL 1730). The synthetic astaxanthin (AstaS) and echinenone standard were purchased from Ehrenstorfer Standards (LGC Standards). Canthaxanthin standard was purchased from Fluka (Buchs, Switzerland).

### 3.2. Algae Material

The powder (AstaP) and AstaCO_2_ oleoresin of *Haematococcus pluvialis*, a microalgae strain from the coast of Chile, were purchased from Pigmentos Naturales (Pigmentos Naturales, SA, Iquique Chile). The extraction of AstaCO_2_ oleoresin was prepared by NATECO2 (85283 Wolnzach, Germany) using supercritical CO_2_ as the solvent for the extraction. This AstaCO_2_ oleoresin is commercially available as 10% w/w AstaCO_2_/sunflower oil. All of those products were stored at −20 °C to avoid degradation of thermal compounds.

### 3.3. Extraction of Natural Astaxanthin

In our study, astaxanthin was extracted in two different ways.

To extract astaxanthin from dried *Haematococcus pluvialis*, encysted cells were crushed then dissolved in DMSO (2 g in 40 mL). The mixture obtained was sonicated (90 s Ultrasonic bath BANDELIN SONOREX RX-100-H) and then stirred for 24 h. After that, a new sonication of 90 s was done, and the solution was centrifuged half an hour at 5251× *g*. The final mixture was filtered (0.2 µm Millipore) and stored at −20 °C. The obtained astaxanthin was called AstaP.

### 3.4. Spectrophotometric Measurements and Astaxanthin Concentration Calculations

Spectrophotometric measurements were made on a Perkin Elmer Lambda 12 Spectrophotometer in the UV-visible domain (200–800 nm), using a scan speed of 50 nm/min. The AstaS spectra show a maximum absorption at 490 nm. A calibration curve was obtained from AstaS.

The astaxanthin extinction coefficient (ε_482_ = 1.25 × 10^5^ L·mol^−1^·cm^−1^) was obtained from the synthetic astaxanthin (AstaS) calibration curve in DMSO and used to calculate the astaxanthin contents in the extracts (AstaP and AstaCO_2_). Our stock solutions for the biological experiments are respectively 2.1 and 127 mM for AstaP and AstaCO_2_.

### 3.5. TEAC

The antioxidant capacities of natural (AstaP), supercritical (AstaCO_2_) and synthetic astaxanthin (AstaS) were realized by the TEAC method [27,28,30]. Briefly, a stock solution of the free radical ABTS^•**+**^ (7 mM) was prepared by mixing vol/vol ABTS solution (7 mM) and potassium persulfate (K_2_S_2_O_8_, 270.322 g/mol, colorless) solution (2.45 mM) in distilled water. The mixture was placed in the dark, at room temperature for 12 to 16 h before use. Then, we diluted the solution in PBS (1/1000) in order to have an absorbance of 0.7 ± 0.2 at 734 nm. The standard antioxidant used in this test was Trolox. Before use, solutions of Trolox and samples were freshly prepared at different concentrations in PBS 1×. Fifty microliters of these solutions were added to 1 mL ABTS^•**+**^ and incubated 1 h at 25 °C; then, absorbance was measured at 734 nm (UV-Visible Lambda 12, PerkinElmer Inc., Norwalk, CT, USA). The inhibition percentage was calculated with the following formula: 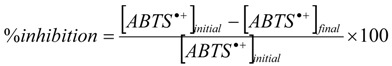


Then, the percentage of inhibition was plotted *versus* the antioxidant concentration. The TEAC corresponds to the Trolox concentration having the same antioxidant activity as the considered sample. To calculate TEAC, we plotted the curves of the inhibition degree of Trolox and of the sample, then we divided the slopes of the curves by the Trolox curve slope. The TEAC coefficient, being a slope ratio, is unitless. We can report this value for the initial mass of products, pure or natural mixtures, in mmol Trolox/g. The TEAC was evaluated at different times.

### 3.6. ORAC

Antioxidant capacity of natural (AstaP), supercritical (AstaCO_2_) and synthetic astaxanthin (AstaS) was performed by the ORAC method [43,44,45]. Briefly, solutions of fluorescein (4 nM), AAPH (2,2′-azobis(2-amidino-propane) dihydrochloride, 160 μM) and Trolox (0–100 μM) were prepared in PBS. One hundred fifty microliters of fluorescein solution were added to each well (96-well microplate), and 25 μL of samples, blank (PBS) or standard (trolox) were placed; then, the reaction was started by adding AAPH (25 μL). Fluorescence was monitored at 37 °C every minute for 60 min (3 × 8 measurements par point) at wavelengths of excitation and emission, respectively, of 485 and 528 nm. The area under the curve (AUC) of relative fluorescence was calculated. ORAC values were expressed as Trolox equivalent in μM and calculated as the slope ratio from curves, AUC_net_, *versus* the concentration of antioxidant and trolox, respectively (AUC_net_ = AUC_sample_ − AUC_blank_).

### 3.7. Chromatography: HPLC-DAD and HPLC-(APCI) Ion Trap MS

Characterization of AstaxP and AstaCO_2_ by HPLC-DAD and HPLC-(APCI+) ion trap MS in the positive ion mode was performed on an Agilent 1100 series (Agilent Technologies, Waldbronn, Germany). The analytical column was an Ultrabase C18 (5 μm, 250 mm × 4.6 mm i.d.) from AnalísisVínicos (Tomelloso, Spain) and kept in a column oven at 20 °C. The mobile phase consisted of an 80-min gradient from 83:17 to 98:2 (acetone/water) at a flow rate of 0.8 mL/min. The injection volume was set at 20 µL. The DAD scan range was from 200 to 800 nm. The MS parameters were: capillary voltage 3500 V, skimmer 40 V, source temperature 400 °C, drying gas 5 L·min^−1^ at 350 °C, nebulizer gas 60 psi, scan range 100–2200 *m*/*z*, maximal accumulation time 200 ms.

### 3.8. MTT Reduction Assay

HUVEC cells were cultured in low glucose (1 g/L) minimum essential medium (MEM) supplemented with 10% (v/v) fetal calf serum, 1% penicillin-streptomycin-amphotericin at 37 °C and 5% CO_2_. Experiments were performed with cells between Passages 10 and 15.

HUVEC cells were seeded in uncoated 24-well cell-culture plates (6 × 10^4^ cells/well) with medium for 48 h. The cells were then treated with various concentrations of astaxanthin (1–15 µM). The final DMSO concentration in the assay in astaxanthin samples never exceeded 1% and had no influence on cell growth. Samples were added at the time of seeding in order to obtain concentrations of 0, 1, 5, 10 and 15 µM. In addition to the control group, we also cultured cells in a medium without sample, but containing 1% DMSO. After 48 h, a total of 1 mL of MTT was added in each well at a concentration of 0.5 mg/mL [46]. The plates were stored for 3 h in the dark at room temperature. Then, MTT solution was removed from each well, and 300 µL of isopropanol were added. Plates were stored once again in the dark and overnight at 4 °C. Optical density (OD) was evaluated at 570 nm on the TECAN plate reader. The test met the acceptance criteria if the mean OD_570_ of blanks was approximately 0.2. If viability was reduced to <70% of the blank, it was considered as having cytotoxic potential [47].

To evaluate cell morphology, HUVEC cells (10^4^ cells/well) were cultured in Lab-Teck plates for 48 h. Then, cells were stained with Alexa Fluor-phalloidin (Molecular Probes) to highlight the cytoskeleton.

### 3.9. Cellular Antioxidant Activity Assay

To measure ROS production in HUVEC cells, we used the DCFH-DA, an oxidation-sensitive indicator. This probe is not fluorescent in its original form and can freely cross cell membranes. Into the living cells, two acetate groups (DA) are removed from the indicator forming DCFH, which is still not fluorescent. In the presence of ROS, DCFH is oxidized to fluorescent 2′,7′-dichlorofluorescein (DCF), which can be measured by fluorometry [48].

HUVECs were cultured to 80% of confluence in low glucose-MEM medium. Then, cells were detached with trypsin and seeded at a density of 10^4^ cells/well in 96-well cell-culture plates. After 24 h of incubation, the medium was washed and DCFH-DA was added for an in-well concentration of 10 µM. Plates were incubated for 1 h at 37 °C. After a full hour of exposition to the probe, the excess of indicator in the medium was washed with PBS, to make sure that only the intracellular oxidation was measured. After the PBS washing, astaxanthin samples were added. Concomitantly, we induced an *in vitro* oxidative stress using the *tert*-butyl hydroperoxide (Luperox, *t*-BuOOH at 100 µM) [49]. Stress controls (without the antioxidant samples and with Luperox) and blanks (without DCFH-DA) were performed on each plate. Fluorescence intensity was measured using a fluorescence spectrophotometer at an excitation wavelength of 485 nm and an emission wavelength of 530 nm. A dose-response curve has been established.

The ROS scavenging activity was expressed as CAA (%) and calculated using the equation: CAA (%) = [(*I*c − *I*s)/*I*c] × 100, described previously [27]. *I*c is the intensity of cells exposed to Luperox without antioxidant sample, and *I*s the intensity of cells exposed to Luperox and samples at different concentrations.

### 3.10. Statistical Analysis

Each experiment was tested three times to determine the reproducibility and to provide the mean ± standard deviation. A *p*-value <0.05 was considered significant. Data were analyzed for statistical significance using one-way analysis of variance (ANOVA), followed by Tukey’s HSD *post hoc* test using JMP software (Version 9; SAS Institute, Cary, NC, USA).

## 4. Conclusions

Astaxanthin has been demonstrated here to inhibit intracellular induced stress in human endothelial cells without any cytotoxicity and modification of the morphology up to 5 μM. However, it should be noted that astaxanthin was dissolved in DMSO, which has itself an influence on the viability of HUVEC cells, representing a limitation in this study. Complementary studies are required to solve the issue of vehicles and/or media for astaxanthin applications.

A comparative study among different astaxanthin sources, extraction methods and final formulations was performed. This study showed that astaxanthin from natural extracts from *Haematococcus pluvialis* had higher antioxidant activity than commercial synthetic astaxanthin. In this context, HPLC-ESI characterization of natural extracts (AstaP and AstaCO_2_) showed the presence of other carotenoids and astaxanthin mono- and di-esters in contrast with synthetic astaxanthin, which contains only a mixture of three isomers of free astaxanthin. Moreover, the final formulation may also have an influence on the antioxidant capacity of astaxanthin. In our study, both AstaCO_2_ and AstaP come from the same source, but they were extracted by using different methods (supercritical fluids and polar solvent, respectively) and presented under different formulations (in sunflower oil and DMSO, respectively). Further studies are necessary to understand the real activity of each fraction of natural extracts.

To conclude, our results strengthen the hypothesis that the antioxidant capacity of astaxanthin contributes to a decrease in the risk of oxidative stress-related diseases, such as cardiovascular diseases.
